# Proceedings of the College of Physicians and Surgeons, of Louisville, on the Death of Dr. Robert J. Breckinridge, Jr.

**Published:** 1867-08

**Authors:** W. B. Caldwell, Coleman Rogers

**Affiliations:** President; Secretary


*Proceedings of the College of Physicians and Surgeons, of Louisville, on the death of* DR. ROBERT J. BRECKINRIDGE, JR.

At a very full meeting of this body, on Monday evening, July 15th, Dr. D. W. Yandell addressed the Fellows as follows:

Since our last meeting this community has been startled by the painful intelligence of the sudden death of one who was long a fellow of this college, and for many years held a high position in his profession in this city. Dr. Robert J. Breckinridge, Jr., died of apoplexy, in Houston, Texas, on the evening of the 8th of July. The mournful duty of making this announcement is, perhaps, justly mine. The deceased and myself were play-fellows when children, were schoolmates and fellow-students at a late period of our lives; subsequently we were professors in the same institution, finally we were surgeons in the same army, and always friends. Not a few of the other members of this college have been associated with Dr. Breckinridge in the medical schools of Louisville; to most he was known personally, and by all he was admired for his sterling qualities of head and heart. Few men were so genial, so social as the deceased; few so gifted by nature, so fitted to shine in his profession. In person he was noble; in manners he was polished, affable, and dignified; his temper was generous, and his sense of honor pure and lofty.

I beg to offer the following resolutions:

*Resolved*, That in the death of Dr. Breckinridge, this College has lost a distinguished Fellow, the medical profession one of its lights and ornaments, and society a member fitted by nature for eminent usefulness.

*Resolved*, That we sincerely condole with the parents and family of the deceased in their deep affliction, and that the Secretary of the College communicate to them a copy of these resolutions.

W. B. CALDWELL, President.

Coleman Rogers, Secretary.

